# Medroxyprogesterone Acetate Decreases Th1, Th17, and Increases Th22 Responses via AHR Signaling Which Could Affect Susceptibility to Infections and Inflammatory Disease

**DOI:** 10.3389/fimmu.2019.00642

**Published:** 2019-04-03

**Authors:** Marie-Pierre Piccinni, Letizia Lombardelli, Federica Logiodice, Ornela Kullolli, Enrico Maggi, Marylynn S. Barkley

**Affiliations:** ^1^Department of Experimental and Clinical Medicine and Center of Excellence for Research, Transfer and High Education DENOTHE of the University of Florence, Florence, Italy; ^2^Immunology Area, IRCCS Bambino Gesù Children's Hospital, Rome, Italy; ^3^Department of Neurobiology, Physiology and Behavior, University of California, Davis, Davis, CA, United States

**Keywords:** hormone replacement therapy, contraception, medroxyprogesterone acetate, Th1, Th2, Th17, Th22, infection

## Abstract

A synthetic progestin, medroxyprogesterone acetate (MPA), was used in a novel study to determine progestin effects on human purified macrophages and Th1, Th2, Th17, Th22 cells. MPA concentrations were equivalent to those in the serum of women after 6 and 9 months of progestin use. MPA has no effect on the proliferation of PBMCs and CD4+ T cell clones induced by immobilized anti-CD3 antibodies or by antigen (streptokinase). However, MPA decreases production and mRNA expression of IL-5, IL-13, IFN-γ, T-bet, RORC, and IL-17A but increases production and mRNA expression of IL-22 by CD4+ Th22 cell clones and decreases IL-22 production by Th17 cells. MPA inhibits RORC, but not T-bet and AHR, by Th17 cells but increases AHR mRNA and T-bet expression of established CD4+ Th22 cell clones. This suggests that MPA, at concentrations equivalent to those found in the serum of women after treatment for contraception and hormone replacement therapy, can directly inhibit Th1 responses (against intracellular bacteria and viruses), Th17 (against extracellular bacteria and fungi), Th2 (against parasites) but MPA therapy increases IL-22 produced by Th22 cells mediated by an increased expression of AHR and T-bet controlling inflammation. MPA could be responsible for the tissue damage limited by IL-22 in absence of IL-17A.

## Introduction

Different CD4+ T helper (Th) lymphocytes have been classified into different functional subsets based on their profile of cytokine production. Type 1 Th (Th1) cells produce interferon-gamma (IFN)-γ, interleukin (IL)-2, and tumor necrosis factor (TNF)-β. They also promote the production of opsonizing and complement-fixing antibodies, macrophage activation, antibody-dependent cell cytotoxicity and delayed type hypersensitivity (1, 2). Type 2 Th (Th2) cells produce IL-4, IL-5, and IL-13 and provide optimal help for humoral immune responses, including IgE isotype switching and mucosal immunity, through mast cell and eosinophil differentiation and facilitation of IgA synthesis. In addition, some Th2-derived cytokines, such as IL-4 and IL-10, inhibit several macrophage functions (1, 2). An additional subset of CD4+T helper cells beyond the traditional Th1 and Th2 cells has been identified more recently, i.e., the Th17 cell, which produces IL-17A, IL-17F, IL-21, IL-26, and IL-22 (3). The major role of Th17 is the protection against extracellular bacteria and fungi. These cells are also pathogenic in several murine models of chronic inflammatory disorders.

Th22 cells primarily secrete IL-22, IL-13, and TNF-alpha. Similar to Th17 cells, Th22 cells express CCR4, and CCR6, but they do not express IL-17, CCL20, IL-23R, CD161 (Th17 markers), IL-4 (Th2 marker), or IFN-gamma (Th1 marker). The expansion of IL-22-producing cells appears to be regulated by the aryl hydrocarbon receptor (AHR) transcription factor, although additional intracellular molecules involved in Th22 differentiation are still being investigated. Expression of the CCR4 and CCR10 skin-homing receptors on Th22 cells suggests these cells are likely recruited to the skin where they may contribute to host defense against microbial pathogens, and promote tissue repair or remodeling. Th22 cells may also be involved in the pathogenesis of inflammatory skin disorders such as psoriasis, atopic eczema, and allergic contact dermatitis (4, 5).

The development of Th1- or Th2-dominated responses depends on several factors, the most critical being cytokines produced in the microenvironment during antigen presentation. The differentiation of Th cells into polarized Th1 or Th2 cells can also be influenced by certain hormones. Some years ago, we showed that progesterone is a potent inducer of helper 2 (Th2) type cytokines (IL-4 and IL-5), leukemia inhibitory factor (LIF) and macrophage colony-stimulating factor (M-CSF) (6, 7). The ability of progesterone to suppress cell-mediated functions via the production of Th2-type cytokines has suggested that the high levels of progesterone in human placenta are of great relevance for the maintenance of pregnancy by promoting the tolerance of the fetus assimilated to an allograft (6–16). Recently, it has been shown that progesterone induces the expression of TSLP (an inducer of Th2-type responses) and inhibits the expression of Th17 related transcription factor RORγt, reducing the influx of neutrophils in murine vaginal gonococcal infection (17).

Synthetic progestins are selected for clinical use primarily to mimic the actions of endogenous progesterone, produced predominantly by the ovaries. The relatively short half-life of the reproductive steroids has, until recently, obviated the therapeutic goal of providing physiological hormone replacement unless parent compounds are pharmaceutically modified to prolong their action. MPA was developed to provide a safe, effective synthetic progestin to women requiring hormone therapy (HRT) and/or contraception. It has selective activity quite similar to progesterone itself (18). As expected, MPA has a more favorable bioavailability and a longer half-life than progesterone. Consequently, we were interested in the possibility that the synthetic progestin, MPA, which mimics progesterone effects could also influence T cell cytokine production.

Medroxyprogesterone acetate (MPA), a 17α-hydroxyprogesterone derivative, is a synthetic analog of the natural steroid progesterone. Since its development more three decades ago, MPA has been employed in the treatment of mammary and endometrial adenocarcinomas (19), as a supportive therapy in the anorexia/cachexia syndrome (20) and is also the most widely used injectable female contraceptive. Known as Depo-Provera, MPA is provided as a long-acting contraceptive (21), with at least 20 million current users worldwide (22). MPA is also the most commonly used progestin in the USA and Europe for hormone replacement therapy (23). The doses of MPA used in humans vary. A single 150 mg intramuscular injection every 3 months (24) creates circulating C_max_ MPA levels ranging from 0.32 to 3.7 ng/ml and C_min_ MPA levels of 0.04 to 1.31 ng/ml (25). After a single injection of 150 mg MPA, time to C_max_ is ~9 days (Package insert, Depo-Subq Provera). Earlier estimates of MPA levels following a single sc injection of this MPA dose are consistent with current measurements: serum concentrations of MPA showed a brief initial elevation ranging from 1.5 to 3 ng/ml for a few days with a gradual decline to about 1 ng/ml for 2–3 months, decreasing gradually to 0.2 ng/ml during the sixth month to < 0.02 ng/ml at about 7.5 to 9 months following administration (26). The blood level of MPA that suppresses ovulation is ~0.1 ng/ml (26). Oral MPA has also been used in contraceptive preparations and most commonly for HRT (10 mg to 1.25 mg daily). The C_max_ for two PREMPRO tablets containing 1.5 mg MPA/tablet is 1.2 ng/ml which is achieved within 2.8 h (Package insert, PREMPRO). Additionally, very high doses of MPA (2,000 mg/day orally) have been used as endocrine therapy for hormone-related cancer (27). The plasma steady-state concentration of MPA with different regimens varied from 1 ng/ml in hormone therapy and contraception to more than 0.2 μg/ml in endocrine treatment of hormone-related cancer.

It is important to note that the scarce information regarding the effects of MPA on immune response has mostly been obtained from patients receiving high dose-schedules (28, 29). Studies carried out in patients receiving high doses of MPA showed that MPA either suppresses or has no effect on lymphocyte proliferation stimulated by mitogens (PHA, Con A) (28–30). High doses of MPA also reduce the production of IL-1β, IL-6, and TNF-α of PHA-stimulated peripheral blood mononuclear cells (PBMCs), providing further evidence that this progestin hinders the activity of cytokines that play a key role in the pathogenesis of the anorexia/cachexia syndrome, also explaining the clinical benefit of MPA treatment in cancer patients with this syndrome (28). It has been shown that blood mononuclear cells cultured in MPA at saturating ligand concentration, 10 μM (from 2 × 10^4^ to 2 × 10^6^ fold higher than in doses found in the serum of MPA users) produced significantly lower levels of IL-1α, IL-12p40, IL-10, IL-13, and G-CSF in response to BCG (31). The authors of this study (31) asserted without showing data that MPA only inhibited IFN-γ production at high but not at concentrations equivalent to serum levels in MPA users (31). Others reported that MPA 10^−6^ M (at doses 1,000 fold higher than those found in the serum of MPA users) inhibited the production of IFN-γ, IL-2, IL-4, IL-6, IL-12, TNF-α, macrophage inflammatory protein-1α (MIP-1α) by peripheral blood mononuclear cells and activated purified CD3+ T (32) MPA also reduced the production of IFN-α and TNF-α by plasmacytoid dendritic cells in response to Toll-like receptor-7,−8, and−9 ligands (32). More recently, using two different murine Mycobacterium tubercolosis models, some authors studied the effect of MPA at doses found in serum of human users. They injected 1 mg/ml of Depot (D) MPA and found concentrations of MPA in the serum of mice from 1 ng/ml to 23 ± 6.90 ng/ml after 1 week and at 0.19 ng/ml after 16 weeks. They reported that DMPA concentrations altered both serum TNF-α, G-CSF and IL-10 in C57BL/6 mice and IFN-γ in BALB/c mice also altering the secretion of IFN-γ, IL-17, GM-CSF, IL-6, and MCP-1 by mononuclear cells from mediastinal lymph nodes stimulated by Mycobacterium tubercolosis antigens (PPD or ESAT6) (33). In mice low concentrations of MPA (10^−9^M and 10^−10^M), more similar to the ones found in the serum of MPA users were unable to induce the secretion of IL-4 and IL-2 by lymph nodes cells and did not exert a proliferating effect on lymph node cells of sheep red blood cells-immunized mice (34), but low dose MPA has the ability in mice to enhance *in vivo* and *in vitro* antibody production (IgM and IgG) (34).

AHR, is an orphan receptor which mediates the effects of a large number of synthetic and natural compounds including halogenated aromatic hydrocarbons like 2,3,7,8-tetrachlorodibenzo-p-dioxin (TCDD) (35). It regulates the expansion of IL-22-producing cells (Th22 and Th17 cells) and is involved in the regulation of a number of physiological processes in many organs, among them all organs of the female reproductive system (36).

Irregular cycles in AHR knockout mice and TCDD-treated rats are evidence for a regulatory function of AHR in the estrous and menstrual cycle (37). Considering that the development and function of the female reproductive system is mainly regulated by estrogens and progestins, a crosstalk between the AHR signaling pathway and sexual steroid hormones is likely. It has been shown that progesterone increases uterine AHR levels in rat endometrial epithelium (35), but apparently MPA does not induce significant changes in AHR transcript levels of endometrial stromal cells (38).

Interestingly, it was been shown that AHR ligands could have different effects on T cell-mediated responses. The AHR ligand TCDD exerts immunosuppressive mediated by AHR effects on the production of IL-2, IL-4, IL-5, and IL-6, whereas M50364, a synthetic compound with antiallergic effects increases IFN-γ but suppresses IL-4 and IL-5 production and the expression of GATA-3, a key transcription factor for Th2 cell differentiation (39). The fact that AHR can act on T helper responses suggested its effects in the development of inflammatory and autoimmune diseases. In fact TCDD administration confers protection from Experimental Autoimmune Encephalomyelitis (EAE), inhibiting Th17 cell differentiation (40). At the time of immunization systemic application of FICZ, another agonist of AHR, also reduced EAE pathology albeit to a lesser degree than TCDD. *In vitro* Th17 differentiation in the presence of AHR agonists, including TCDD, promoted IL-17 and IL-22 expression, by Th17 cells but did not induce Treg differentiation.

The role of MPA on human lymphocyte function has been investigated *1)* at higher concentrations of MPA than those found in the serum of MPA users (28) and, 2) on heterogeneous populations of peripheral blood and lymph node mononuclear cells (28, 31, 32, 34, 41). The observed effects of MPA on the supposed lymphocytes could be mediated by cytokines produced by a cell type present in the mononuclear cell fraction in response to MPA and not by the direct effect of MPA on T cells. We designed a study to examine the direct effect of MPA on human T CD4+ cells at concentrations equivalent to those found in serum of MPA users from 6 months to 9 months following administration [from 0.2 to 0.02 ng/ml (28)]. We determined the effect of MPA on the proliferation, production and mRNA expression of IFN-γ, IL-5, IL-10, IL-4, IL17, and IL-22 of human established CD4+ T cell clones, which cannot be contaminated by other cells present in the PBMC fractions and on Th2-, Th1-, Th22, and Th17-specific transcription factors (GATA 3, T-bet, AHR, ROR-C, respectively) mRNA expression. For the first time the effect of MPA on IL-22 and AHR expression by T helper cell subpopulations has been investigated.

## Materials and Methods

All the methods used for the study were performed in accordance with the relevant guidelines and regulations.

### Donors

Twenty-seven healthy donors of peripheral blood agreed to participate to the study at AOU Careggi, Florence, Italy. They received verbal and written information about the aim and the design of the research, and all donors signed the informed consent and the study was approved by local ethic committee of AOU Careggi (n.115303). The 27 donors (age mean ± SD; 29.9 ± 0.9 years) were male (14) (mean age ± SD; 30.5 ± 4.2 years) and female (13) (mean age ± SD; 29.8 ± 4.1 years). There were no significant age differences between the groups of male and female donors. Donors who were enrolled had normal body BMI and had negative results for illnesses (infections, autoimmune and inflammatory diseases), exposure to communicable diseases, travel to disease endemic areas, pregnancy and lactation, medical, and surgical interventions, history of recent infections, currently under the influence of alcohol or drugs, or undergoing therapy with hormonal or anti-inflammatory therapy in particular.

## Reagents

PHA was purchased from GIBCO Laboratories (Grand Island, N.Y.) and phorbol 12-myristate 13-acetate (PMA) from Sigma Chemical Co. (St. Louis, MO). OKT3 (anti-CD3) mAb was purchased from Ortho Pharmaceuticals (Raritan, N.J.). Anti-CD4, anti-CD8 were obtained from Becton-Dickinson (Mountain View, Ca). Human recombinant IL-2 was a generous gift of Eurocetus (Milano, Italy). Human recombinant IL-12 was obtained from RD systems (Minneapolis, MN). FCS was from HyClone Lab Inc. (Logan, UT). 3H-thymidine was from Amersham (Buckinghamshire, UK). Highly purified MPA (6α-methyl-17α-hydroxyprogesterone acetate) and Mifepristone were purchased from Sigma Aldrich (St. Louis, MO). Streptokinase (SK) was purchased from Aventis Behring GmbH (Germany).

### Generation of T Cell Clones

To generate T-cell clones, peripheral blood mononuclear cells (PBMCs) of normal subjects were seeded under limiting dilution conditions (0.3 cell/well) in 6 round-bottomed microwell plates containing 10^5^ irradiated (9,000 rad) allogeneic PBMC (as feeder cells) and PHA (1% vol/vol) in a final vol of 0.2 ml complete medium supplemented with IL-2 (50 U/ml) and 10% FCS, as reported elsewhere (6). Growing microcultures were then supplemented, at weekly intervals, with IL-2 (50 U/ml) and 10^5^ irradiated feeder cells. The phenotype distribution of T-cell clones was assessed by flow cytometer analysis.

### Proliferation of T Cell Clones Stimulated by Immobilized Anti-CD3 Antibodies in the Absence and in the Presence of MPA

2 × 10^5^ T cell blasts obtained from 7 CD4+ T cell clones, able to produce IL-4, IL-5, IL-13, IL-10, IFN-γ, IL-17A, IL-17F, IL-22, in 0.2 ml RPMI 1640 medium supplemented with 2 mM L-glutamine, 2 × 10^−5^ M 2-mercaptoethanol and 10% FCS (Hyclone Laboratories Inc., Logan, UT) (complete medium) were stimulated in 96 U-bottomed plates with immobilized anti-CD3 antibodies in the absence or in the presence of MPA (0.02, 0.2 and 2 ng/ml) for 5 days. These concentrations were chosen on the basis of those found in the serum during contraception and HRT (26). After a 16-h pulse with 0.5 μCi 3H-TdR (Amersham International), cultures were harvested and radioactivity measured by liquid scintillation.

### Proliferation of PBMCs Stimulated by SK in the Absence and in the Presence of MPA

2 × 10^5^ PBMCs of 5 donors in 0.2 ml of complete medium in 96 U-bottomed plates were stimulated for 5 days with PHA and the antigen Streptokinase (SK) (500 U.I./ml) in the absence or in the presence of MPA (0.02, 0.2, and 2 ng/ml) for 5 days. After a 16-h pulse with 0.5 μCi 3H-TdR (Amersham International), cultures were harvested and radioactivity measured by liquid scintillation.

### Induction of Cytokine Production by T Cell Clones in the Absence and in the Presence of MPA

To induce cytokine production, 2 × 10^5^ T blasts from each 23 CD4+ T cell clone were cultured in the presence of immobilized anti-CD3 mAb (10 μg/ml) in the absence or the presence of MPA (0.02 and 0.2 ng/ml). After 36 h, culture supernatants were collected, filtered, and stored in aliquots at −70°C until used. For mRNA determination the cells were collected after 6 h.

### Induction of the Cytokine Production of PBMCs Stimulated by SK in the Absence and in the Presence of MPA

2 × 10^5^ PBMCs obtained from 15 donors in 0.2 ml of complete medium in 96 U-bottomed plates for 5 days were stimulated with the antigen (SK 500 U.I./ml) in the absence or in the presence of MPA (0.02 and 0.2 ng/ml). After 5 days supernatants were collected and stored in aliquots at −80°C until used.

### Induction of Cytokine Production of Macrophages Stimulated by SK in the Absence and in the Presence of MPA

10^6^ monocytes obtained from 7 donors were purified by adherence from PBMCs (mean ± SD; 92.59 ± 2.51%) in 1 ml of complete medium in 96 U-bottomed plates for 5 days with the antigen SK (10 μg/ml) in the absence or in the presence of MPA (0.02, 0.2, and 2 ng/ml). After 5 days supernatants were collected and stored in aliquots at −80°C until used.

### Total RNA Extraction and Real Time Quantitative RT-PCR IL-4, IL-13, IL-5, IFN-γ, GATA-3, T-bet, IL-17, IL-22, ROR-C, AHR, and β-actin

Total RNA was extracted with RNAsy Kit and treated with DNase I (Qiagen, Hilden Germany) from 5 CD4+ T cell clones, and PBMC from 5 donors. cDNA was synthetized by using TaqMan Reverse Transcription Reagents (Applied Biosystem, Warrington, United Kingdom). Reverse Transcription–Polymerase Chain Reaction (RT-PCR) was then performed by using TaqMan methodology as described elsewhere (42). Quantitative analysis of IL-4, IFN-γ, IL-10, IL-13, IL-5, GATA-3, T-bet, IL-17, IL-22, ROR-C, AHR, and β-actin was performed by using Assay on Demand (Applied Biosystem, Warrington, United Kingdom). β-actin was used for normalization.

### Quantitation of Cytokine Production by CD4+ T Cell Clones

The quantitative determination of IL-1beta, IL-1RA, IL-2, IL-4, IL-5, IL-6, IL-8, IL-9, IL-10, IL-12, IL-13, IL-15, IL-17A, IFN-alpha, TNF-alpha, G-CSF, GM-CSF, VEGF, PDGF, FGF, IP-10, MCP-1, RANTES, eotaxin, MIP-1-alpha, and MIP-1-beta was performed by a bead-based multiplex immunoassay (Biorad Laboratories, Hercules, CA). IL-17A, IL-17F, IL-21 and IL-22 were measured by a bead-based multiplex immunoassay (R&D, Minneapolis, MN) and a Bioplex 200 system (Biorad Laboratories, Hercules, CA) as we previously described (43, 44).

### Statistical Analysis

Statistical analyses were performed using SPSS software (SPSS, Inc, Evanston, IL). Due to non-parametric distribution, all comparisons between cytokine concentrations in basal and stimulated conditions were performed by Wilcoxon test. Data are reported as median and ranges unless otherwise stated.

## Results

### Effect of MPA on the Proliferation and on the Cytokine Profile of Peripheral Blood Mononuclear Cells (PBMCs)

We used PBMCs to mimic *in vitro* a T cell specific response to an antigen derived from a pathogen. We investigated the effect of MPA when the antigen presenting cells in the PBMC fraction present an antigen to T cells and activate these T cells. Streptokinase (SK), a highly purified antigen extracted from a C-group beta-hemolytic streptococci culture and devoid of all other metabolic products of streptococci, was used as antigen.

Unstimulated PBMCs from 5 normal donors cultured in the presence of MPA at 0.02, 0.2, and 2 ng/ml showed no significant differences in their proliferative response compared PBMCs cultured in medium alone (data not shown).

Streptokinase (SK)- stimulated PBMCs from 5 donors in the presence of MPA at 0.02, 0.2, and 2 ng/ml showed no significant differences in their proliferative response compared to SK-stimulated PBMCs expanded in the absence of MPA (data not shown).

**Figure 1:**
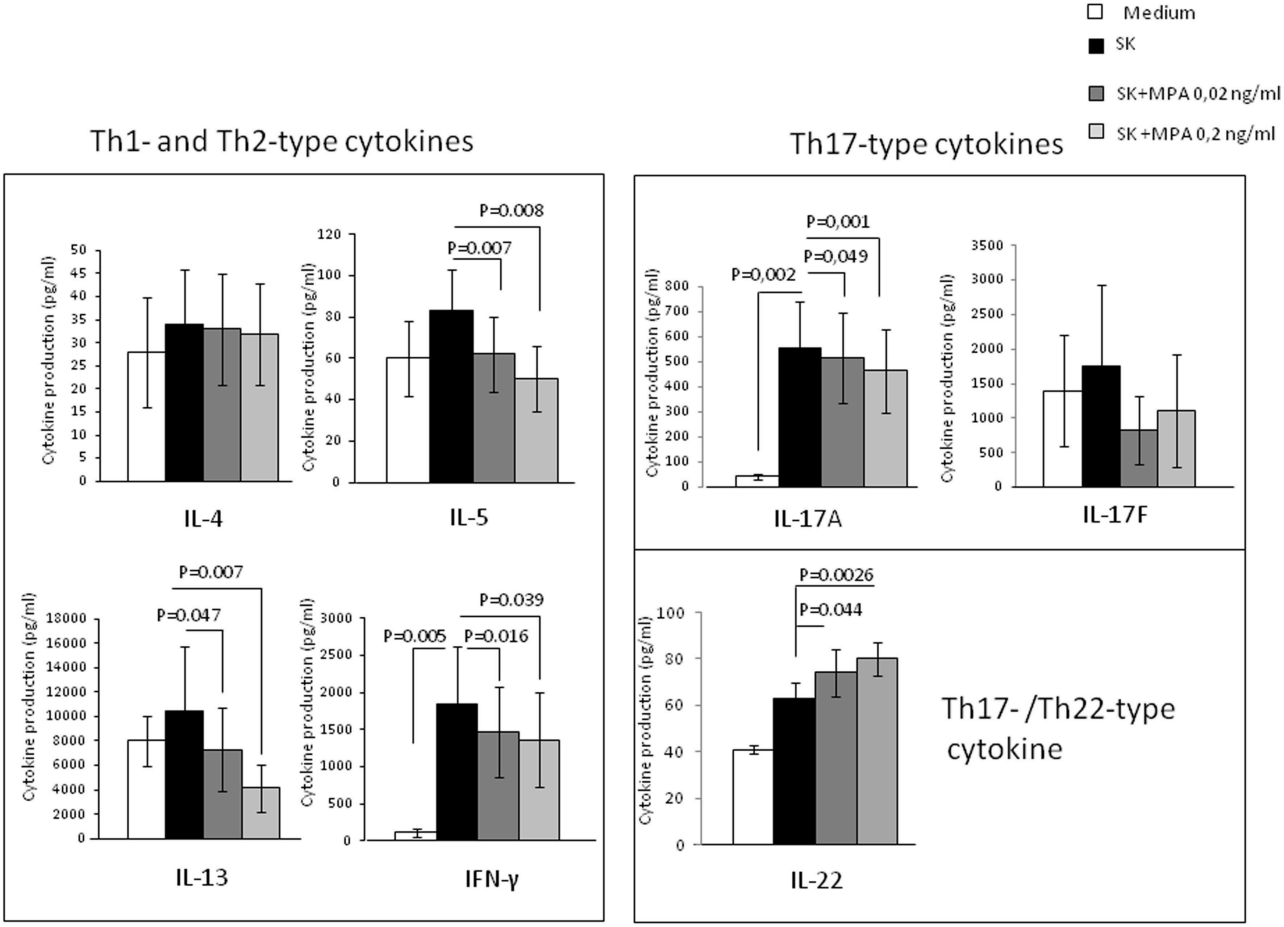
Effect of MPA on the cytokine profile of peripheral blood mononuclear cells (PBMCs). PBMCs from 10 different donors were stimulated with SK in the absence or presence of MPA at 0.02 and 0.2 ng/ml to provide their ability to modulate Th2-type cytokine (IL-4, IL-5, IL-13), Th1-type cytokine (IFN-gamma) and Th17-type cytokines (IL-17A, IL-17F, and IL-22) production.

To provide evidence of the effect of MPA on PBMCs, we analyzed the ability of MPA to act on Th2-type cytokines (IL-4, IL-5, and IL-13), Th1-type cytokine (IFN-gamma) and Th17-type cytokines (IL-17A, IL-17F, and IL-22) production (Figure 1) by PBMCs from 10 different donors. There was no statistical difference in PBMCs cytokine production when cells were cultured without any stimulation in medium alone and in medium + MPA at 0.02, 0.2, and 2 ng/ml (data not shown).

The effects of SK stimulation alone over medium were not statistically significant for IL-4, IL-5, IL-13, IL-22, and IL-17F production in PBMCs, but significant for IFN-γ and IL-17A (Figure 1).

When PBMCs were stimulated with SK in the absence or presence of MPA, the levels of IFN-γ, IL-5, IL-13, IL-17A were significantly decreased whereas the levels of IL-22 were significantly increased in the presence of MPA compared to those found in the absence of MPA (Figure 1), indicating that MPA seems to modulate T cell cytokine production regardless the amplitude of the effect of SK alone. Thus, MPA seems to modulate the T cell cytokine production only after the stimulation of T cells by an antigen (here SK) presented by the antigen presenting cells in the PBMCs fraction.

**Figure 2:**
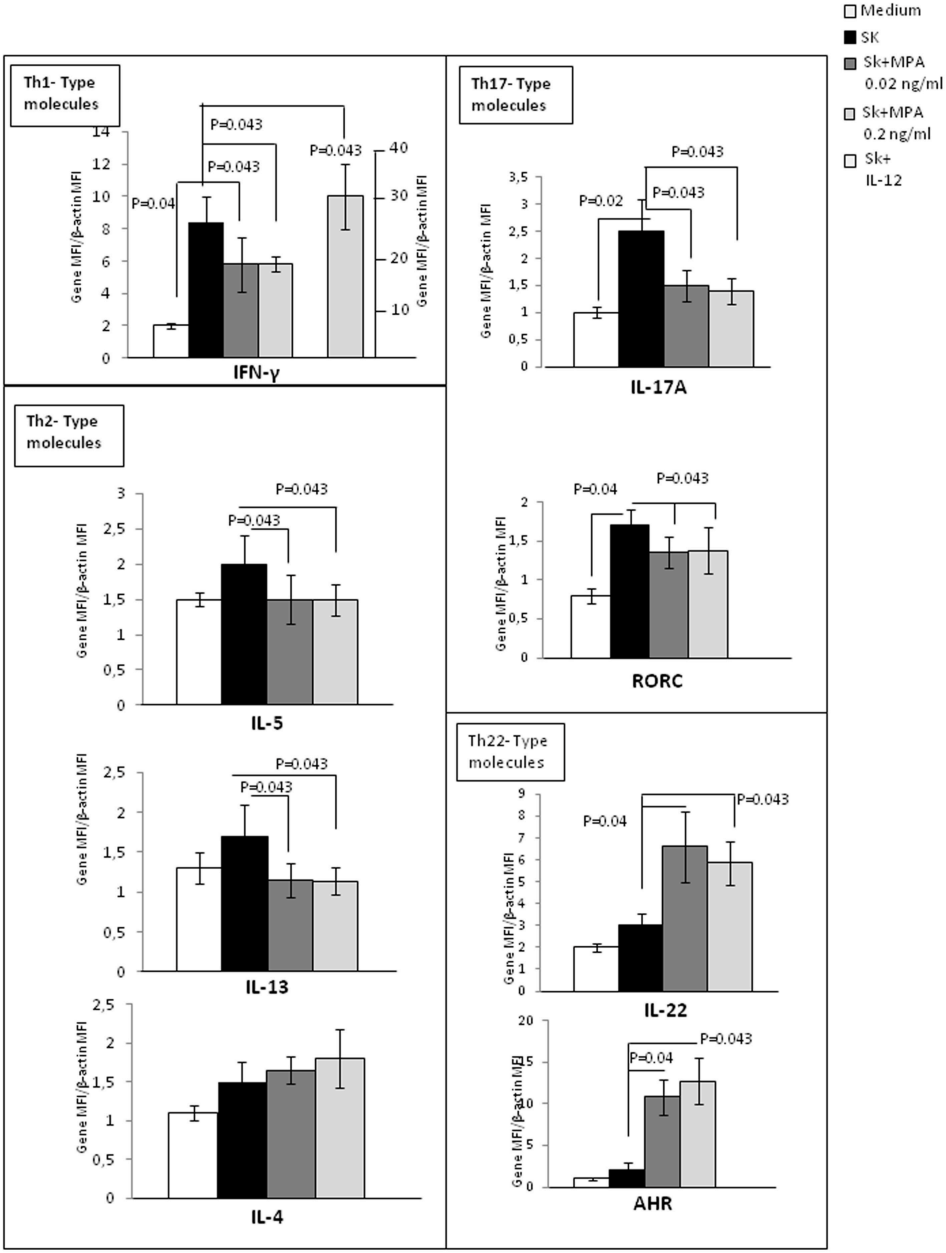
Effect of MPA on the cytokine profile and transcription factor expression by peripheral blood mononuclear cells (PBMCs). mRNA expression of IL-4, IL-5, IL-13, IL-17A, IFN-γ, IL-22, AHR, and RORC performed by RT-PCR analysis of the PBMCs from 5 donors stimulated with SK in the absence or in the presence of 0.02 and 0.2 ng/ml of MPA was analyzed. PBMCs were also stimulated with SK in the presence of IL-12, which is a potent inducer of Th1 differentiation, to ensure that the culture conditions were satisfactory for modulation of the cytokine production of CD4+ T cell clones.

We also attempted to confirm the previous results by examining with real time RT-PCR analysis of PBMCs of 5 additional donors stimulated with SK in the absence or in the presence of 0.02 and 0.2 ng/ml of MPA (Figure 2). As a control, the PBMCs were also stimulated with SK in the presence of IL-12, a potent inducer of Th1 differentiation (45). We found a significant increase of IFN-γ (*p* = 0.043) (Figure 2) in response to IL-12, suggesting that the culture conditions were satisfactory for the modulation of the PBMCs. PBMCs expressed lower levels of mRNA for IL-5, IL-13, IFN-γ, IL-17A and its transcription factor ROR-C (Figure 2). Higher levels of mRNA were found for IL-22 and the corresponding transcription factor AHR (Figure 2) when MPA was added to the culture medium. mRNA levels for IL-4 were not modified by MPA (Figure 2).

These results indicate that MPA can decrease some Th2-type cytokines (IL-5 and IL-13, but not IL-4), including a Th1 cytokine (IFN-γ) and a Th17-type cytokine (IL-17A). However, MPA increases IL-22, which can be produced by Th22 and Th17 cells.

### Effect of MPA on the Cytokine Profile of Macrophages Derived From Peripheral Blood Monocytes

**Figure 3:**
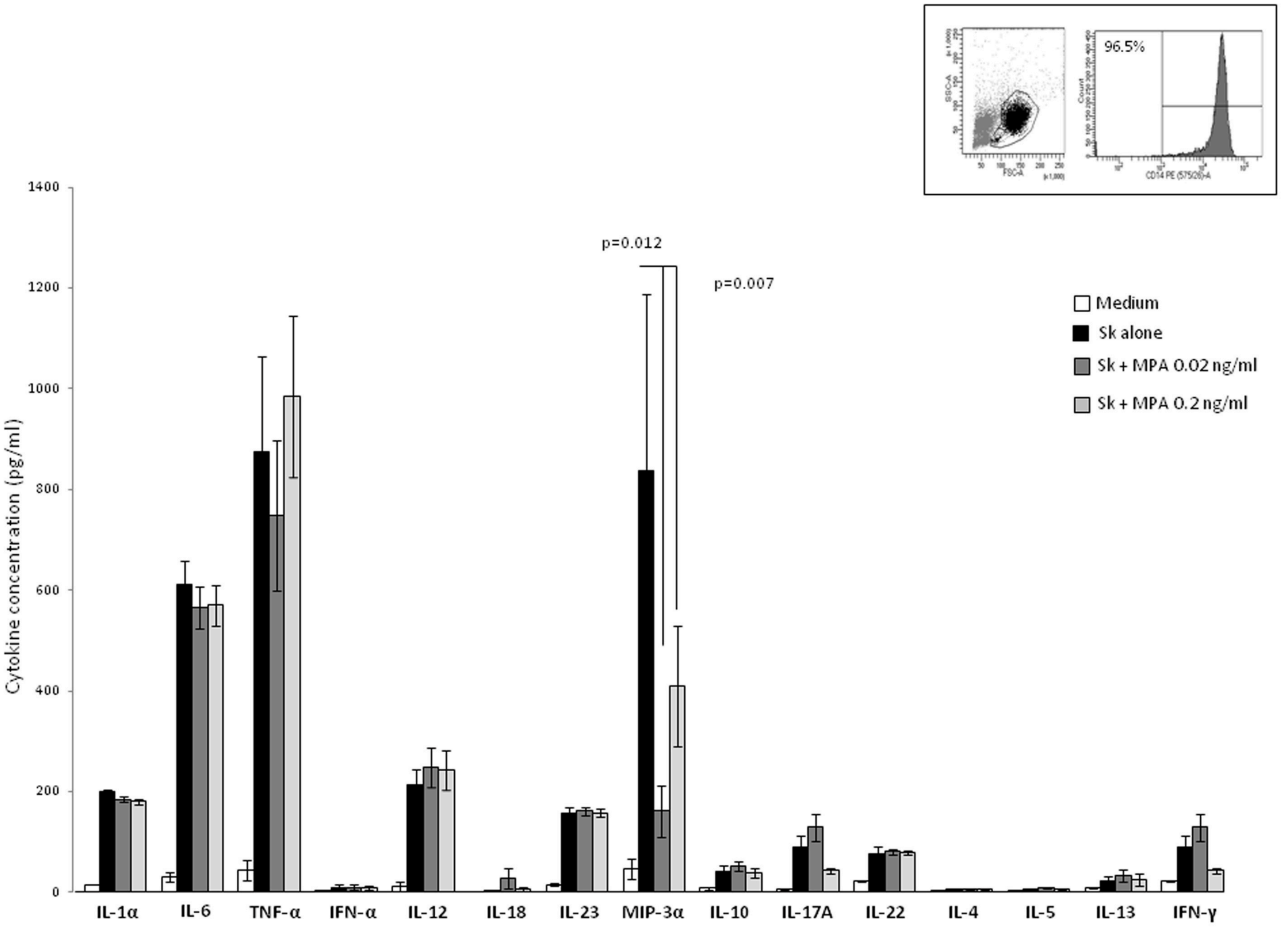
Effect of MPA on the cytokine profile of macrophage. Macrophages from PBMC obtained from 7 donors purified by adherence stimulated for 5 days with the antigen SK in the absence or in the presence of MPA (0.02, 0.2, and 2 ng/ml). IL-1α, IL-6, TNF-α, IFN-α, IL-12, IL-18, IL-23, MIP3α, IL-10, IL-4, IL-5, IL-13 IL-17A IFN-γF, and IL-22 were measured in the supernatants.

The negative effect of MPA on Th1, Th2, Th17-type cytokine production of PBMCs and its positive effect on Th22-type cytokine production could be due to the modulating effect of MPA on APCs present in the microenvironment of the T cells. However, the levels of these cytokines produced by SK-stimulated macrophages cultured in the presence of MPA were not significantly different than those of SK-stimulated macrophages cultured in the absence of MPA (Figure 3). Thus, in PBMC fractions the negative effect of MPA on Th1, Th2, Th17-type cytokine production of PBMCs and its positive effect on Th22-type cytokine production seem to be due to the action of MPA directly on T cells.

Moreover, cytokine production by macrophages in response to MPA could in turn modulate the T cell cytokine production. The negative effect of MPA on T cell-IFN-γ-production was not due to a decreased production of IL-12 and/or IFN-α and/or IL-18 by macrophages treated with MPA, i.e., the concentrations of IL-12, IFN-α, TNF-α, and IL-18 produced by purified macrophages from 7 different donors stimulated with SK showed no significant differences when cells were cultured either in the absence or in the presence of MPA (**Figure 5**). Thus, MPA acts directly on CD4+ T cells to decrease IFN-γ production and mRNA expression (Figures 1, 2). However, the levels of IL-1α and IL-6 produced by SK-stimulated macrophages cultured in the presence of MPA (2 ng/ml) (data not shown) and the levels of MIP-3α (CCL20) in the presence of MPA at 0.02, 0.2, ng/ml (Figure 3) and 2 ng/ml (data not shown) were lower than those of SK-stimulated macrophages cultured in the absence of MPA. The decreased production of IL-1α and IL-6 by macrophages in response to MPA at 2 ng/ml, i.e., at a concentration higher than found in the serum of women treated with MPA after 6 and 9 months, was reported at 200 ng /ml (28). MIP-3α is the known ligand of CCR6, which is expressed on the cell membrane of Th17 cells. The interaction CCR6/CCL20 contribute to the trafficking of Th17 cells. Thus, the reduction of IL-17 production by T cells in the presence of MPA at doses found in the serum of human users could be associated with a reduction of the trafficking of Th17 cells. The levels of IL-23 essential for development of Th17, produced by SK-stimulated macrophages were not modified by the presence of MPA suggesting that MPA acts directly on CD4+ T cells by decreasing IL-17 production.

### Direct Effects of MPA on the Proliferation and on the Cytokine Profile of Established CD4+ T-Cell Clones

To investigate the direct effect of MPA on the proliferative activity of unstimuled purified T cells, 7 T cell clones in medium alone and in medium plus MPA at 0.02, 0.2 and 2 ng /ml were analyzed for their ability to proliferate. No statistical difference of the T cell clones proliferative response was found when cells were cultured without any stimulation in medium alone and in medium plus MPA at 0.02, 0.2, and 2 ng/ml (data not shown).

The direct effect of MPA on the proliferative activity of CD4+T cell clones stimulated with immobilized anti-CD3 antibody in the absence or presence of MPA at 0.02, 0.2, and 2 ng/ml was analyzed. According to the results obtained with PBMCs, no significant differences in the proliferative response were observed between stimulated T cell clones expanded in the presence or in the absence of MPA (data not shown).

**Figure 4:**
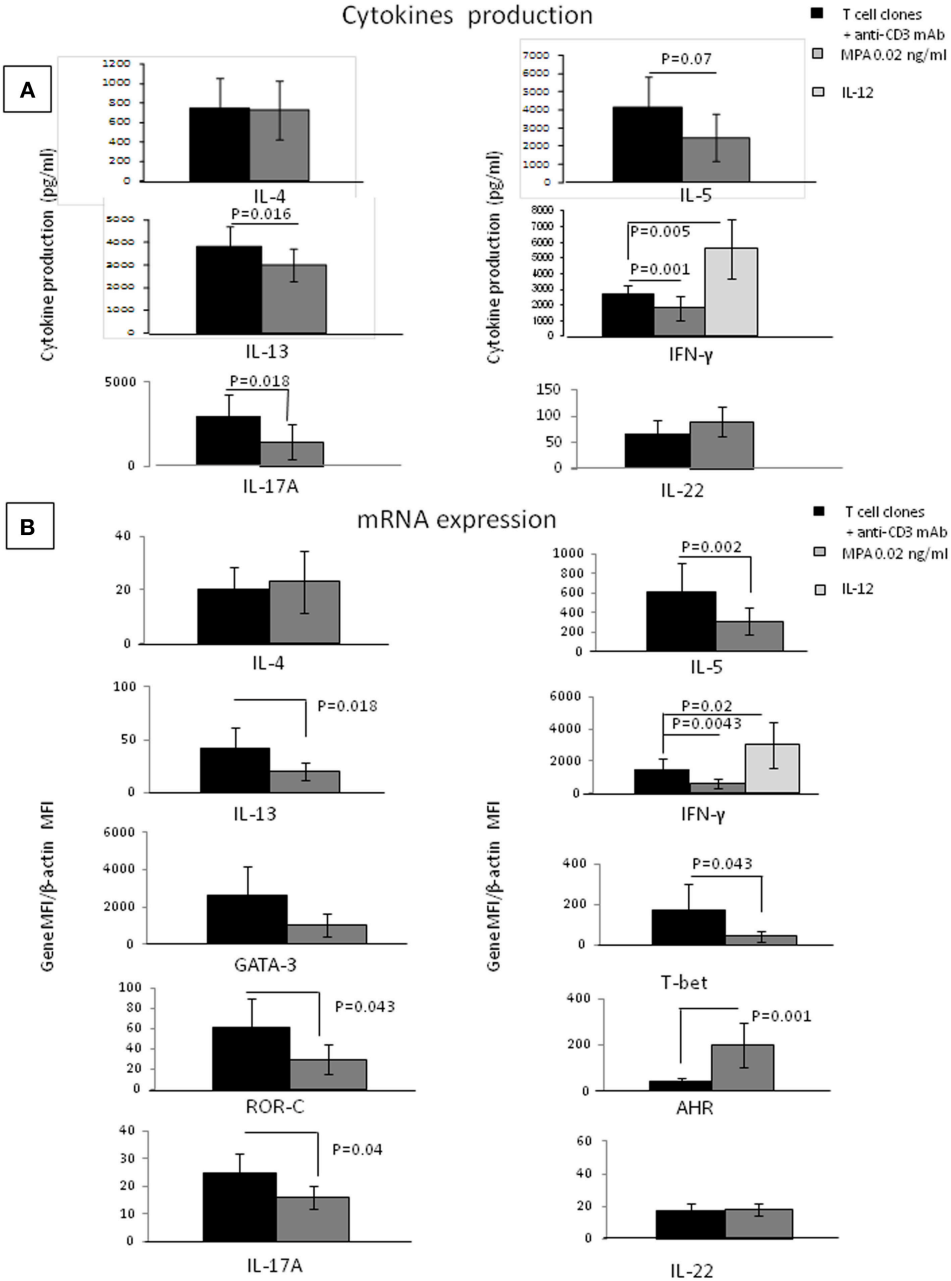
Direct effects of MPA on the cytokine profile of established CD4+ T-cell clones. IL-4, IL-5, IL-13, IL-17A, IFN-γ, IL-17F, and IL-22 production were measured in the supernatant of 13 established T-cell clones (TCC) by multiplex assays **(A)**. mRNA expression for IFN-γ, IL-5, IL-4, IL-13, IL-17A, IL-22, GATA3, T-bet, and AHR was examined **(B)**. As a control, the 13 CD4+ T cell clones were also stimulated with immobilized anti-CD3 antibodies in the presence of IL-12, a potent inducer of Th1.

To provide evidence of the direct effect of MPA on CD4+T helper cells, we examined the ability of MPA to act on a panel of cytokines (as listed in Methods)^.^ We also studied MPA effects on IFN-γ, IL-5, IL-4, IL-13, IL-17A, IL-22, and IL-10 mRNA expression by established CD4+ T-cell clones. Thirteen CD4+ T-cell clones were cultured in the presence of recombinant human IL-12, a Th1 inducer, which acts as a control of the functional activity of T cell clones. When the 13 CD4+ T-cell clones were cultured with IL-12, the levels of IFN-γ and mRNA for IFN-γ increased (Figures 4A,B) (*p* = 0.02 and *p* = 0.005, respectively) suggesting that the culture conditions were satisfactory for the modulation of CD4+ T cell clones.

There was no statistical difference between the T cell clones cytokine production when cells were cultured without any stimulation in medium alone or in medium plus MPA at 0.02, 0.2, and 2 ng/ml (data not shown).

When Thirteen CD4+ T-cell clones were cultured in the absence of any other cell type and stimulated with insolubilized anti-CD3 monoclonal antibody in the absence or presence of MPA the levels of IL-1beta, IL-1RA, IL-2, IL-6, IL-8, IL-9, IL-10, IL-12, IL-15, IL-17F, TNF-α, G-CSF, GM-CSF, VEGF, PDGF, FGF, IP-10, MCP-1, RANTES, eotaxin, MIP-1-α, MIP-1-β, IL-21 (data not shown), IL-22, and IL-4 (Figure 4A), were not significantly modified in the presence of MPA. In contrast, the levels of IFN-γ, IL-5, IL-13, IL-17A were significantly decreased in the presence of MPA compared to those found in the absence of MPA (Figure 4A). Thus, MPA seems to modulate the T cell cytokine production of T cells only when these cells are stimulated.

Real time RT-PCR analysis was used to determine MPA influence on IFN-γ, IL-5, IL-13, IL-17A, IL-4, IL-22, GATA-3 (a Th2-specific transcription factor) (46, 47) T-bet (a Th1-restricted transcription factor) (48), ROR-C (a Th17-specific transcription factor) and AHR (a transcription factor expressed by Th22 cells) mRNA expression of the CD4+T cell clones stimulated with immobilized anti-CD3 monoclonal antibody without or with MPA (Figure 4B). CD4+ T cell clones were also stimulated with immobilized anti-CD3 antibodies in the presence of IL-12. We confirmed an increase of IFN-γ in response to IL-12, suggesting that the culture conditions were satisfactory for the modulation of CD4+ T cell clones (Figure 4B). The CD4+ T cell clones expressed lower levels of IFN-γ, IL-5, IL-13, IL-17A, ROR-C, and T-bet mRNA in the presence of MPA compared to the control, whereas the levels of transcripts for IL-4 and GATA-3 were not modified by the presence of MPA in the culture but the levels of AHR increased (Figure 4B).

Surprisingly, we found that the levels of IL-22 were increased when MPA is added to cultures of PBMCs, but not when it is added to the CD4+ T cell clones, although the levels of m RNA for AHR the transcription factor of Th22 cells producing IL-22 expressed by the same T cell clones were increased.

This apparent paradox could be explained by the fact that IL-22 is not only produced by Th22 cells, but also by Th17 cells, and there could be a differential production of IL-22 by Th17 and Th22 cells in response to MPA. AHR could be the key factor explaining the effect of MPA on the T cells. To investigate this possibility, we studied the effects of MPA on the cytokine profile of established CD4+ Th1- Th2-Th-17 and Th22-cell clones.

### Direct Effects of MPA on the Cytokine Profile of Established CD4+ Th1- Th2-Th17 and Th22-Cell Clones

**Figure 5:**
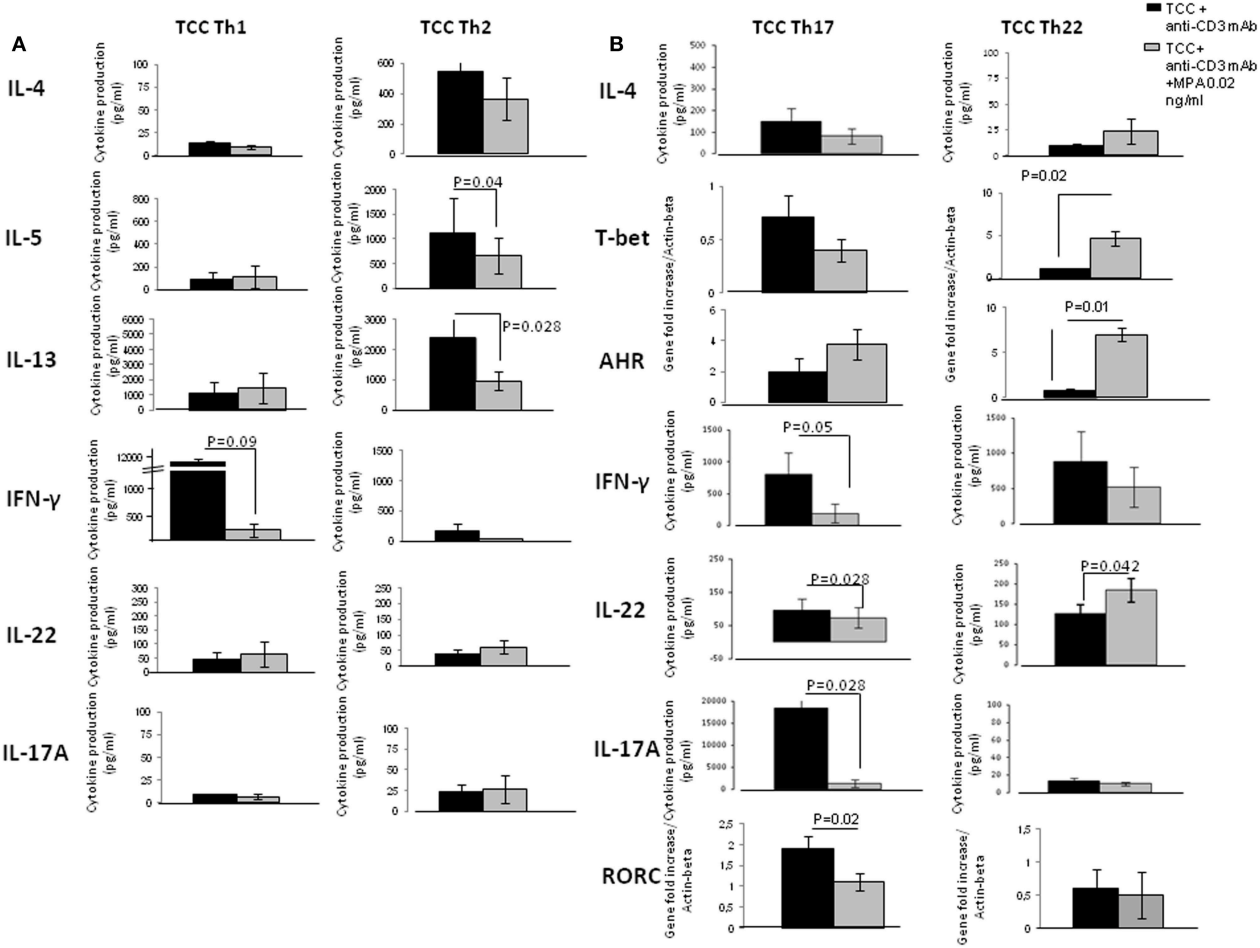
Direct effects of MPA on the cytokine profile of established CD4+ Th1- Th2-Th-17 and Th22-type T cell clones (TCC). To provide evidence of the direct effect of MPA on different CD4+T cell subpopulations, 6 CD4+ Th1 T-cell clones, 6 CD4+ Th2 T-cell clones, 6 CD4+ Th17/Th1 T-cell clones and 6 CD4+ Th22 T-cell clones were stimulated with insolubilized anti-CD3 monoclonal antibody in the absence or presence of MPA at 0.2 ng/ml. The levels of IL-4, IL-5, IL-13, IL-17A, IFN-γ, and IL-22 were measured by multiplex assays in the supernatants **(A)** and of mRNA for AHR, RORC, and Tbet by RT-PCR **(B)**.

To verify the direct effect of MPA on the different CD4+T cell subpopulations, 6 Th1 clones, 6 Th2 clones, 6 Th17/Th1 clones and 6 Th22 clones were stimulated with insolubilized anti-CD3 monoclonal antibody in the absence or presence of MPA (Figures 5A,B) and at protein level analyzed for IL-4, IL-5, IL-13, IL-22, IL-17A, IFN-γ. mRNA levels were determined for AHR, ROR-C, and T-bet.

**Figure 6:**
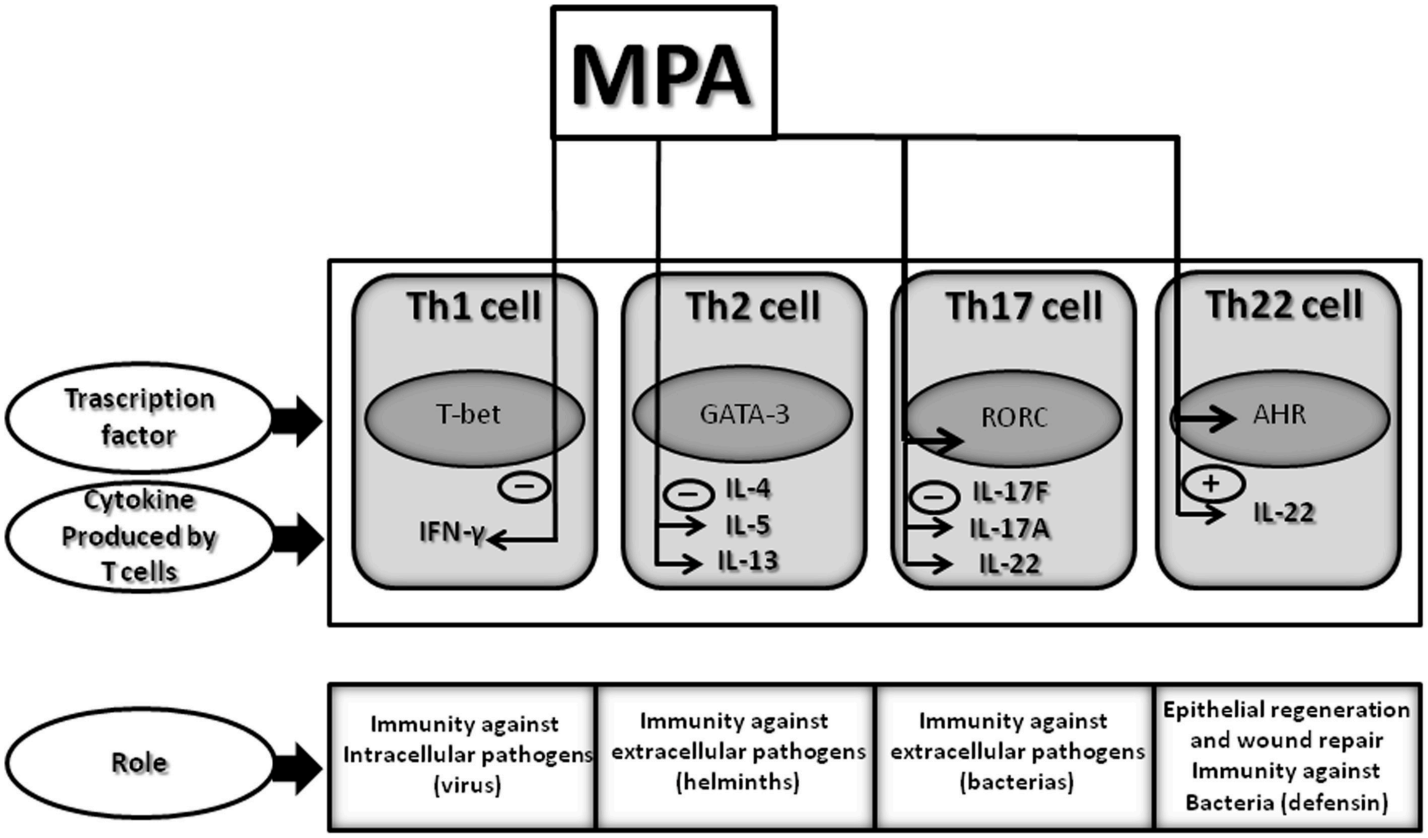
Effect of MPA on the cytokine profile of established CD4+ Th1, Th2, Th17, and Th22 cells: summary of identified changes and potential outcomes. Therapeutic concentrations of MPA, comparable to those present in the serum of women undergoing HRT or contraception, decreases IFN-γ, IL-5, IL-13, and IL-17A production but increases IL-22 production by CD4+ T cells. Thus, MPA by decreasing IFN-γ by Th1 cells could diminish the T cell immune responses to intracellular pathogens (viruses); by decreasing IL-5, MPA could diminish the T cell immune responses to helminths; by decreasing IL-13 could affect allergic disorders; by decreasing IL-17A, MPA could diminish the T cell immune responses to extracellular pathogens; and by increasing IL-22 by Th22 cells MPA may contribute to the host defense against microbial pathogens, in addition to promoting tissue repair or remodeling.

In the presence of MPA levels of IL-4, IL-5, IL-13, IL-22, IL-17A produced by Th1 cells were not significantly modified. In contrast, the levels of IFN-γ produced by Th1 cells were significantly decreased in the presence of MPA compared to those found in the absence of MPA (Figure 5A).

The levels of IL-4, IFN-γ, IL-22, IL-17A produced by the Th2 cells were not significantly modified in the presence of MPA. However, the levels of IL-5 and IL-13 produced by Th2 cells were significantly decreased in the presence of MPA compared to those found in the absence of MPA (Figure 5A).

The production of IL-4 (data not shown), IL-5 (data not shown), IL-13, AHR and T-bet mRNA expression by Th17/Th1 cells were not significantly modified in the presence of MPA. Levels of IFN-γ, IL-22 and IL-17A at protein and mRNA levels (data not shown), and mRNA for ROR-C expressed by Th17/Th1 cells were significantly decreased in the presence of MPA compared to those found in the absence of MPA (Figure 5B).

The levels of IL-4, IL-5 (data not shown), IL-13 (data not shown), IFN-γ and IL-17A produced by the Th22 cells were not modified whereas the levels of mRNA for AHR, T-bet and IL-22, as well as levels of IL-22 protein (*p* = 0.042) were significantly increased in the presence of MPA compared to those found in the absence of MPA. Moreover, the levels of mRNA for ROR-C were not modified in Th22 cells in presence of MPA compared to those found in absence of MPA (Figure 5B).

These data confirmed the negative effects of MPA on the production of IFN-γ, IL-5, IL-13, and IL-17A, but supported the finding that Th17 and Th22 cells, which are able to produce IL-22, react differently to MPA; IL-22 production in response to MPA is increased in Th22 cells and decreased in Th17 cells. This could explain why the statistical analysis of IL-22 production by 13 different T- cell clones derived from different Th type subpopulations does not appear to be influenced by MPA (Figure 4A). The increased production of IL-22 by Th22 cells is probably compensated by the decreased production of IL-22 by the Th17 cells in response to MPA. Moreover, it seems that T-bet and AHR control the production of IL-22 by Th22.

## Discussion

We found that therapeutic concentrations of MPA, comparable to those present in the serum of women undergoing HRT or contraception, have no effect on the proliferation of CD4+ cells and several cytokines, (IL-1beta, IL-1Ra, IL-2, IL-4, IL-6, IL-8, IL-9, IL-10, IL-12, IL-15, IL-17F). Neither do these doses of MPA affect TNF-α, G-CSF, GM-CSF, VEGF, PDGF, FGF, IP-10, MCP-1, RANTES, eotaxin, MIP-1α, MIP-1β or IL-21 production by CD4+T cells. In contrast, MPA specifically decreases IFN-γ, IL-5, IL-13 and IL-17A production but increases IL-22 production by CD4+ T cells. These results were confirmed by examining MPA effects on mRNA levels. Furthermore, MPA decreased not only IL-17A production by Th17 cells but also MIP-3α (CCL20) by macrophages, which contributes to decreased trafficking of Th17 cells.

Interestingly, IL-22 production is decreased in Th17 cells treated with MPA but increased in Th22 cells. The decreased production of IL-17A and IFN-γ in the presence of MPA is associated with the decreased expression of ROR-C and T-bet, respectively, whereas the increased production of IL-22 by Th22 cells is associated with the increased expression of T-bet and AHR in response to MPA.

Thus, MPA, at doses equivalent to those found in the serum of women using MPA for contraception or HRT, could influence T cell immune responses toward allograft rejection, infections and asthma as demonstrated *in vivo* in humans (49–56).

Indeed, an allograft rejection is characterized by a predominant production of IFN-γ (57, 58). Our results showing the depressed production of IFN-γ in response to MPA are consistent with data from animal model studies indicating that MPA prolongs survival of skin and renal allografts (49, 59, 60). More interestingly, in a trial of humans with intrafamilial renal transplants treated by addition of MPA to the immunosuppressive regimen, when renal function was stable at the time of initiation of MPA therapy, prednisone dosage could be lowered to an average of 37% of the previous dose (49). Thus, MPA has been shown to have immunosuppressive functions in human.

IFN-γ also plays an important role in adaptive and innate immune responses to viral and intracellular bacterial infection (61). The decreased IFN-γ production by T cells in response to therapeutic concentrations of MPA suggests this synthetic progestin could influence antiviral immunity. In fact, MPA is used to increase infectibility in mouse models of sexually transmitted diseases (62). Relative to progesterone, MPA was shown in these models to increase by ten-fold susceptibility to infection by Herpes simplex virus type 2 (HSV-2) (62). Unlike progesterone, MPA significantly decreases the immune response to intracellular pathogens (63). A Th1 response seems to be protective against HSV viral spread and tissue damage, but shifting to a Th2 response is associated with resolution of immunopathology (64, 65). MPA, by decreasing IFN-γ production by T cells, could be, at least in part, responsible for an increased susceptibility to infection by Herpes simplex virus. More importantly, it was reported that consistent MPA contraceptive use in 682 HSV2-negative women induced increased risk of HSV-2 seroconversion. In fact, incidence rate was 13.5 per 100 person-years in women consistently using DMPA (nine incident infections per 66.5 person-years) and 6.6 per 100 person-years in women who were neither pregnant nor using hormonal contraception (35 incident infections per 529.5 person-years) (52). Very recently, an updated systematic review incorporate studies published between January 2009 and June 2017. Thirty articles met the inclusion criteria and showed that Depo-medroxyprogesterone acetate (DMPA) increased the risk of HSV-2 (strong effect, few studies), whereas data on oral contraceptive use suggested it was associated with inconclusive findings for HSV-2 (53).

A Th1/Th2 cytokine imbalance is also critical to HIV-1 progression and pathogenesis. It was shown that addition of exogenous IFN-γ mediates antiviral activity against R5 HIV-1 thymocytes, which decreases viral replication in infected thymocytes (66). It was suggested that MPA suppresses both innate and adaptive arms of the immune system resulting in a reduction of host resistance to invading pathogens as HIV-1 (32). However, MPA was used in culture at doses 1,000 fold higher than the those found in the serum of MPA users. In the present study we show for the first time that doses of MPA equivalent to those found in women actually using MPA decrease IFN-γ production by T cells, which could influence HIV progression. Thus, MPA, by increasing shedding of HIV-1 DNA (67), increases the HIV transmission risk, and, by decreasing IFN-γ production by T cells, may also influence HIV viral replication. There is now a major concern in women who use long acting injectable hormonal contraceptives, particularly Depo-MPA with an increase of HIV-1 risk acquisition. In fact, different recent meta-analyses showed a significant association between use of Depo-MPA and the presence or acquisition of HIV+ in women (54–56). For this reason, the World Health Organization (WHO) published guidelines for hormonal contraceptive eligibility for women at high risk of HIV in March 2017. The guideline development group, through a consensus, made recommendations to change the medical eligibility criteria for contraceptive use from category 1 (condition for which there is no restriction for the use of the contraceptive method) to category 2 (condition where the advantages of using the method generally outweigh the theoretical or proven risks) for Depo-MPA among women at high risk of HIV acquisition (68).

It is important to recognize that MPA is the most commonly used progestin in the USA and Europe for hormone replacement therapy (23). In addition, MPA is the most widely used injectable female contraceptive (21), with at least 20 million current users worldwide (22). Of special interest in the developing world, with its high incidence of viral diseases and endemic malnutrition, we suggest that the choice of synthetic progestins used in contraception could have important implications for viral disease development. It is important to note, however, that the use of hormonal contraceptives, and in particular of MPA, does not significantly impact the effectiveness of antiretroviral therapy (25, 69).

As progesterone can induce an increased production of IL-5 and IL-4 by T cells, yet have no effect on T cell IFN-γ production (6), MPA is the only progestin yet analyzed that influences IFN-γ production by Th1 cells when used as a contraceptive or for HRT and also decreases IL-5 and IL-13 production without affecting IL-4 production. As a result of this activity MPA may also have an influence on allergic responses. Allergy is a disorder characterized by an increased ability of B cells to produce IgE in response to certain groups of ubiquitous antigens (allergens) that can activate the immune system after inhalation, ingestion or penetration through the skin. IgE antibody synthesis results from the collaboration between Th2 cells and B cells, in which CD40/CD40L interaction is required (70). IL-4 and IL-13 produced by Th2 cells induce the production of IgE by B cells whereas Th1 cells produce IFN-γ that suppresses IgE synthesis (71). Th2 cells also produce IL-5 that favors the differentiation, activation and survival *in situ* of eosinophils. MPA by acting to decrease IL-5, IL-13 and IFN-γ production by T cells could have a negative effect on the differentiation of eosinophils, their activation and survival while having a positive effect on IgE production induced by IL-4. The latter activity would decrease the negative effect of IFN-γ on the IgE production. Asthma is a complex disorder characterized by intermittent, reversible airway obstruction, and by airway hyperresponsiveness and inflammation. Asthma may be divided into allergic (extrinsic) and non-allergic (intrinsic) asthma. Both allergic and non-allergic asthma are characterized by the presence in the bronchial mucosa of large numbers of activated eosinophils and of elevated concentrations of eosinophil-derived proteins, such as major basic protein and eosinophil cationic protein (72). In allergic asthma, the importance of Th2 cytokines, especially IL-5, in the induction of allergic pulmonary inflammation and airway hyperreactivity has been reported (73). Corticosteroid treatment in asthma, is associated in the downregulation of BAL cells expressing mRNA for IL-4 and IL-5 and in the upregulation of cells expressing mRNA for IFN-γ (74). MPA decreases IL-5 and IL-13 production and mRNA expression by T cells. This suggests that widely used concentrations of MPA can have a positive influence on the health status of patients with allergic and non-allergic asthma by decreasing IL-5 production by T cells, thus reducing eosinophilic infiltration of the lungs. Interestingly, after 6 months of HRT with transdermal 17-beta-estradiol and MPA, diminishing symptoms of asthma were observed and there was a reduction in the number of patients in whom it was necessary to use oral glucocorticoid therapy during exacerbation of asthma (50, 51). These patients were treated with 17-beta-estradiol associated with MPA. However, the diminishing symptoms of asthma cannot be due to the estradiol, which potentiates the development of asthma. In fact, in male mice treated with estradiol, eosinophil numbers increase in both blood and airways and the production of IL-5 and IL-13 by T cells is promoted (75). Thus, estradiol increases IL-5 produced by T cells, whereas MPA decreases IL-5. Therefore, the reduced asthmatic symptoms in patients treated with 17-beta-estradiol and MPA could be due to the reduction of IL-5 production mediated by MPA.

We found that MPA at therapeutic concentrations found in the serum of women upregulates AHR. AHR plays important physiological roles in many cells of the immune system, notably the Th17 and Th22 cells (76–78). Most of the current literature on AHR effects on immune system function is focused on the consequences of exposure to the high affinity ligand 2, 3, 7, 8 tetrachlorodibenzo-p-dioxin (TCDD). However, many recent studies in mice lacking AHR expression indicate that AHR activation affects important physiological functions in the absence of xenobiotic ligands (79). There is a range of potential physiological ligands for AHR including diet-derived AHR ligands (Quercetin present in apples and onions, Indol-3-carbinol present in many Brassicaceae, Resveratrol present in red wine, Curcumin), which strongly influence intestinal immune parameters (79). Our data seem to suggest that MPA could be another ligand for AHR. In agreement with the hypothesis that steroid hormones could affect AHR expression and could be a ligand of AHR, it was demonstrated that progesterone, as well as 17-beta-estradiol, regulate the AHR battery homeostasis in the rat uterus (37). Progesterone leads to an increase in uterine AHR levels, especially in endometrial epithelium. Only one demonstration of MPA influence on endometrial but not T cells showed that no significant changes were observed in AHR transcript levels in endometrial cells (35). While these results indicate that female sexual steroid hormones regulate the expression of the AHR battery in organs of the female reproductive system, no effectiveness of female hormones, in particular MPA, on the expression of the AHR battery in T cells has been previously reported.

The present study show that MPA, at concentrations found in the serum of women undergoing contraception or HRT, up-regulates IL-22 production by CD4+ Th22 cells through AHR and T-bet -induced signals, whereas IL-22 production by Th17 cells is down-regulated and Tbet and AHR are not modified by MPA. These data suggest that the differential production of IL-22 by Th22 cells and Th17 cells by MPA could be carried out through AHR-induced signals and T-bet-induced signals. In fact, it appears that IL-22 expression is due to the cooperation of AHR and T-bet-induced signals (78).

The ligand dependant-AHR activity is involved in the regulation of T cell-mediated immune responses (76, 77) and, as such, could be involved in shaping the course of autoimmune pathology. This suggest a link to environmental factors containing ligands of AHR that influence autoimmune disease. AHR-deficient mice developed a much milder form of EAE with many of these mice altogether protected from the onset of disease (76). However, the application of AHR agonists caused differential effects. The administration of the tryptophan metabolite 6-formylindolo(3,2-b) carbazole (FICZ), an endogenous AHR agonist, exacerbated disease (76), while systemic administration of TCDD had the same ameliorating effect on disease progression as AHR-deficiency (77). This led to the suggestion that AHR exerts its effects on immune responses in a ligand-dependent manner. The differential effects of different AHR agonists on autoimmune disease progression is probably due to their influence on the T helper responses responsible for the disease progression (39). Indeed, it has been demonstrated that the oral administration of a synthetic compound M50354, a AHR agonist, reduces the production of IL-4 and IL-5 by Ag-stimulated splenocytes and enhances the production of IFN-γ. TCDD, another AHR ligand, exerts suppressive effects on the production of IL-2, IL-4, IL-5, and IL-6. In contrast, M50367 did not affect the production of IL-2 and IL-6, but appeared to reduce Th2-mediated immune responses. M5037 suppressed the expression of a key transcription factor for Th2 cell differentiation, GATA-3, and the production of IL-4, although it is not known whether activated AHR is directly involved in GATA-3 expression (39).

These data suggest that AHR may play an important role in the normal development and function of the immune system by down-regulating IFN-γ. In agreement, we showed that MPA has no effect on IL-4, upregulates AHR, downregulates IFN-γ production by T cells. Moreover, it was shown that in response to OVA immunization, high levels of IFN-γ mRNA were detected in lymphocytes from AHR Knock-out (AHR-/-) mice, but IL-4 mRNA levels in AHR-/- cells were similar to those in AHR+/+ mice (80).

The effects of AHR agonists on IL-22 production have been reported (81). AHR is down-regulated in intestinal tissue of patients with IBD; and AHR signaling via IL-22 inhibits inflammation and colitis in the gastrointestinal tract of mice (81). Intestinal lamina propria mononuclear cells in the presence of FICZ showed reduced levels of IFN-γ and up-regulated levels of IL-22 (81). We showed that MPA downregulates IFN-γ and upregulates AHR and IL-22 expression and production by T cells as does FICZ, the agonist of AHR.

Our results allow us to speculate that MPA could be an agonist of AHR in T cells, acting to decrease IFN-γ and, at the same time, IL-17A, upregulating IL-22 without any effect on IL-4 production.

It is important to note that AHR acts as an important co-factor in infections. For instance, AHR-deficient mice infected with Listeria monocytogenes, an intracellular bacterium, were more susceptible to infection but developed enhanced resistance to re-infection (82). Depending on the cell context analyzed and type of agonist used, AHR-driven signals could exert differential modulation of Th responses and act as initiators or attenuators of tissue- damage T cell-dependent inflammatory processes.

Through the AHR expression MPA, may potentially decrease immune function by decreasing T cell IFN-γ and IL-17A production, thereby influencing certain aspects of infection progression. By increasing IL-22 and decreasing IL-17A, MPA may be tissue protective (83). IL-22 induced by MPA could act as a protective hormone thereby counteracting the destructive effects of the immunoresponse. MPA may also limit the tissue damage observed in some autoimmune disorders, and may attenuate the inflammatory processes (81) in some autoimmune disorders.

The biology and pharmacology of progestins and their receptors are complex. Our understanding of their action in certain physiologic targets including the immune system continues to grow. The specific effects of hormones, including synthetic progestins, depend upon their preparation, dose, sequence of administration and context of treatment. The observation that ubquitously used doses of MPA influence the immune response of women adds yet another level of complexity to the design and prescription of hormone therapy, whether it be contraception or HRT, particularly in women with coexisting immune disorders and infections.

## Author Contributions

M-PP conceived the study and designed the experiments, analyzed all the data, supervised, and wrote the manuscript. MB participated in discussion and revision of the manuscript. LL, FL, and OK performed the experiments using PBMNCs and T cell clones cultures, performed the multiplex bead-based assays and RT-PCR. EM approved and authorized all the process.

### Conflict of Interest Statement

The authors declare that the research was conducted in the absence of any commercial or financial relationships that could be construed as a potential conflict of interest.
